# Phytochemical and Antioxidant Investigation of the Aerial Parts of *Dorema glabrum *Fisch. & C.A. Mey.

**Published:** 2015

**Authors:** Mohammad-Reza Delnavazi, Abbas Hadjiakhoondi, Abbas Delazar, Yousef Ajani, Saeed Tavakoli, Narguess Yassa

**Affiliations:** a *Department of Pharmacognosy, Medicinal Plant Research Center, Faculty of Pharmacy, Tehran University of Medical Sciences, Tehran, Iran.*; b *Department of Pharmacognosy, Faculty of Pharmacy, Tabriz University of Medical Sciences, Tabriz, Iran.*

**Keywords:** *Doremaglabrum*, Apiaceae, Elemicin, Myristicin, Flavonoid, Caffeoylquinic acid, DPPH

## Abstract

*Dorema glabrum *Fisch. & C.A. Mey. (Apiaceae) is a monocarpic perennial plant distributed in southern Caucasus. In Azerbaijan Republic folk medicine, the gum-resin of this species is used as a diuretic and anti-diarrheal agent. It is also traditionally used for the treatment of bronchitis and catarrh. In the present study, chemical constituents of the essential oil and extract of *D. glabrum *aerial parts were investigated and their free radical scavenging potentials were assessed. GC-MS and GC-FID analyses of the plant essential oil resulted in identifying twenty compounds, out of which elemicin (38.6%) and myristicin (14.3%) were main compounds. Seven compounds including daucosterol (1), chlorogenic acid (2), a mixture of cynarin (3) and 3,5-di-O-caffeoylquinic acid (4), isorhamnetin-3-O-β-D-glucopyranoside (5), isoquercetin (6) and astragalin (7) were also isolated from the ethyl acetate and methanol fractions of *D. glabrum *aerial parts using different chromatographic methods on silica gel (normal and reversed-phase) and sephadex LH_20_. Structures of the isolated compounds were elucidated using UV and ^1^H, ^13^C-NMR spectrain comparison with those reported in respective published data. Antioxidant activities of the crude extract, fractions and isolated compounds were evaluated using DPPH free radical scavenging assay method. Among the fractions, methanol fraction (IC_50_=53.3 ±4.7μg mL^-1^) and among the isolated compounds, caffeoylquinic acid derivatives exhibited the highest free radical scavenging activity (IC_50_= 2.2-2.6 μg mL^-1^).

## Introduction

The genus *Dorema *D. Don of the Apiaceae (*alt.* Umbelliferae) family comprises 12 species, mainly distributed in southwestern and central Asia (1, 2). In Iran this genus is represented by seven species, and *Dorema glabrum *Fisch. & C.A. Mey. is one of them (3).

*D. glabrum*is a monocarpic perennial plant native to the northwest of Iran, Azerbaijan Republic (in Nakhichevan region) and Armenia (2, 3). Like other species of the genus *Dorema*, *D. glabrum* exudes a gum-resin which is used in Azerbaijan Republic folk medicine as a diuretic and anti-diarrheal agent as well as for the treatment of bronchitis and catarrh (4).There are also some reports indicating that this plant is used by indigenous people for cure of some cancer types (5).

Previous pharmacological investigations have shown hypocholesterolemic and antioxidant activities of *D. glabrum* aerial parts and anti-proliferative effects of its fruits against WEHI-164 mouse fibrosarcoma cell line (6, 7). The essential oil of *D. glabrum* roots has also been reported to contain δ-cadinene (12.8%) and *β-*bisabolene (7.5%) as main compounds with a week antioxidant activity in DPPH free radical scavenging assay (IC_50_= 2.2 mg mL^-1^) (5). Unfortunately, extensive exploitation of this medicinal plant and reduction of its natural population has led it to be considered as an endangered species (8).

The present study was an attempt to investigate the phytochemical constituents and antioxidant properties of the aerial parts of *D. glabrum*. To our knowledge, this is the first report onessential oil composition and isolation of compounds with free radical scavenging activity from the aerial parts of this medicinal species. 

## Experimental

*Plant material*

The aerial parts of *D. glabrum* were collected from "Ghaflankuh" mountains located in East-Azerbaijan (northwest of Iran) during its flowering stage in June 2011. The voucher specimen of the plant (voucher no. 1640-IMPH) was deposited at the herbarium of Institute of Medicinal Plants, ACECR, Karaj, Iran.

*Essential oil extraction*

The air-dried and comminuted plant material (100 g) was subjected to hydrodistillation for 4 h using a Clevenger-type apparatus to produce essential oil with 0.2% yield. The obtained oil was dried over anhydrous sodium sulfate and stored in 4 °C until analysis. 

*GC-MS and GC-FID analyses*

GC-MS analyses of the essential oil were carried out on a Hewlett-Packard 6890 gas chromatograph with fused silica HP-5MS column (30 m ×0.25 mm I.D., 0.25 μm film thickness) coupled with a mass detector (Hewlett-Packard model 5973 HP). The flow rate of carrier gas (Helium) was 1 mL min^-1^. The initial oven temperature was 40 °C and was then raised to 250 °C at a rate of 3 °C min^-1^. The injection temperature was 250 °C and the oil sample (1 μL) was injected with a split ratio of 1:90. The mass spectra were obtained by electron ionization at 70 eV. The retention indices (RI) of the compounds were calculated using a homologous series of n-alkanes injected in conditions equal to the samples. Identification of the compounds were based on computer matching with the Wiley7n.L library, direct comparison of the retention indices and fragmentation pattern of the mass spectra with those for standard compounds data published in the literature (9).

Relative percentages amounts of the identified compounds were achieved using an Agilent HP-6890 gas chromatograph equipped with a FID detector. The FID detector temperature was 290 °C and the operation was performed under the same conditions as described above for GC-MS analyses.

*Extraction and fractionation*

The air-dried and ground aerial parts (0.8 Kg) were macerated with methanol (4 L ×5) at the room temperature. The obtained crude extract (180 g) was moved on a silicagel column (30-75 mesh, Merck, Germany) and eluted successively with petroleum ether, chloroform, ethyl acetate and methanol (each 4 L), to get four main fractions. All the fractions were concentrated under the maximum temperature 45 °C using a rotary evaporator.

*Phytochemical analyses*

In preliminary studies by thin layer chromatography using various reagents (TLC, Pre-coated Silica gel GF254 and silica gel 60 RP-C18 F254s plates, Merck, Germany), ethyl acetate and methanol fractions were found to contain number of spots characteristic for phenolic compounds. These fractions were then subjected to more phytochemical investigation using various chromatographic and spectroscopic methods.

Ethyl acetate fraction (20 g) was moved on a silica gel column (230-400 mesh, Merck, Germany) and eluted with MeOH-CHCl_3_(0.5:9.5 - 3:7)to give nine fractions (E_1-9_). Fraction E_3 _afforded 120 mg of white crystals which were purified on a silica gel column (MeOH-CHCl_3_, 1:9) to get compound 1(80 mg).Chromatography of the fraction E_8_ (1.2 g) on a sephadex LH_20_ (Fluka, Switzerland) column, eluting with MeOH, yielded to seven fractions (E_8a-8g_). Fraction E_8g_ (32 mg) was chromatographed over silica gel column, eluting with[H_2_O-HCO_2_H-CH_3_CO_2_H-EtOAc (2.4:1:1:63)] to get compound 2(14 mg). A portion of methanol fraction (4 g) was moved on an RP-C18 (230-400 mesh, fully endcapped, Fluka, Switzerland) columnand eluted with a gradient mixture of CH_3_CN-H_2_O (1:9 - 3:7) to yield ten fractions (M_1-10_). Fraction M_7_ (43 mg) was chromatographed overa C18 reversed-phase column (CH_3_CN-H_2_O, 1.5:8.5) to get a mixture of compounds 3 and 4 (12 mg). Compound 5(22 mg) obtained from the fraction M_9 _(51 mg) by chromatography on a sephadex LH_20_ column, eluted withMeOH-H_2_O (8:2). Reversed-phase chromatography of the fraction M_10_ (50 mg) on an RP-C18 column (CH_3_CN-H_2_O, 2:8) afforded compound 6 (7 mg) and 7 (8 mg). These compounds (7 and 8) were then further purified on sephadex LH_20_ column, eluted with MeOH-H_2_O (8:2).

In all steps, column chromatography was monitored by TLC under UV at 254 and 366 nm and by spraying anisaldehyde-H_2_SO_4 _reagent followed by heating (120 °C for 5 min) and the fractions giving similar spots were then combined. 

The structures of compounds were determined by UV spectrophotometer (CE7250, Cecil) using various shift reagents (10), ^1^H-NMR,^13^C-NMR and DEPT spectral analyses [Brucker Avance 400 DRX (400 MHz for ^1^H and 100 MHz for ^13^C)] as well as by comparison with respective published data.

*DPPH free radical scavenging activity*

The crude extract, fractions and isolated compounds were evaluated for their free radical scavenging activities using 2, 2-diphenyl-1-picryl-hydrazyl (DPPH) method described by Sarker *et al*. with slight modifications (11). Briefly, the prepared sample solution (5 mg mL^-1^) in methanol was serially diluted to get concentrations ranging from 0.5 to 9.5×10^-3^ mg ml^-‌‌1^. Diluted solutions (1 mL each) were mixed with 1 mL of DPPH (Sigma-Aldrich, Germany) solution (80 μg mL^-1^ in methanol) and were kept 30 min at 25 °C in dark for any reaction to take place. 

UV absorbances of the mixtures were recorded on a Cecil CE7250 spectrophotometer at 517 nm. Butylated hydroxytoluene (BHT) was also used as a positive control. All tests were performed in triplicate and IC_50_ values were reported as means ±SD.

## Results and Discussion

Twenty compounds, representing 88.8% of the oil, were identified as a result of GC-MS and GC-FID analyses of the oil obtained from the aerial parts of *D. glabrum*.The results showed that the tested oil was rich in oxygenated non-terpenes (56.3%) with elemicin (38.6%) and myristicin (14.3%) as main compounds (Table 1).

**Table 1:** Chemical composition of the essential oil of *D. glabrum *aerial parts

**No.**	**Compounds** a	**RI** b	**Methods of identification**	**%**
1	α-Pinene	939	MS/RI	1.5
2	2-Pentyl furan	984	MS	2.1
3	β-Myrcene	988	MS/RI	0.4
4	Limonene	1024	MS/RI	0.4
5	-β-Ocimene(Z)	1032	MS/RI	0.4
6	-β-Ocimene(E)	1044	MS/RI	1.0
7	Fenchyl acetate	1229	MS/RI	0.6
8	Tridecane	1300	MS	0.8
9	Ylangene	1373	MS/RI	1.2
10	β-Cedrene	1419	MS/RI	4.1
11	Geranyl acetone	1453	MS/RI	1.2
12	β-Chamigrene	1476	MS/RI	1.6
13	Cuparene	1504	MS/RI	5.0
14	Myristicin	1517	MS/RI	14.3
15	β-Bisabolene	1529	MS/RI	7.7
16	Calacorene	1544	MS/RI	0.9
17	Elemicin	1555	MS/RI	38.6
18	Germacrene B	1559	MS/RI	1.7
19	Nerolidol	1561	MS/RI	4.0
20	Tetradecanal	1611	MS/RI	1.3
				
	Hydrocarbonemonoterpenes			3.7
	Hydrocarbonesesquiterpenes			22.2
	Hydrocarbone non-terpenes			0.8
	Oxygenated monoterpenes			1.8
	Oxygenated sesquiterpenes			4.0
	Oxygenated non-terpenes			56.3
	Unidentified			11.2
	Total identified			88.8

a Note: Compounds listed in order of elution from HP-5MS column;

b Retention indices to C_8_–C_24_ n-alkanes on HP-5MS column.

A review of the previous studies on essential oil of *Dorema *species including *D. ammoniacum *and *D. aucheri *aerial parts and* D. glabrum *roots, showed that elemicin and myristicin have not been detected in their chemical composition and therefore, this is the first report on identification of these phenylpropanoid derivatives in the essential oil of *Dorema* genus plants (5, 12, 13, 14, 15).Elemicin and myristicin, however, have been reported at high levels in essential oils of* Ferula *species, the genus which is classified in the same tribe (Scandiaceae) as *Dorema* genuse (16, 17).

Considering to the reported antifungal and antibacterial effects of elemicin and hepatoprotective, anti-inflammatory and insecticidal properties of myristicin, the essential oil of *D. glabrum* aerial parts might also possess similar pharmacological potentials through notable amounts of these two bioactive compounds in its chemical content (18-22).

**Figure 1:**
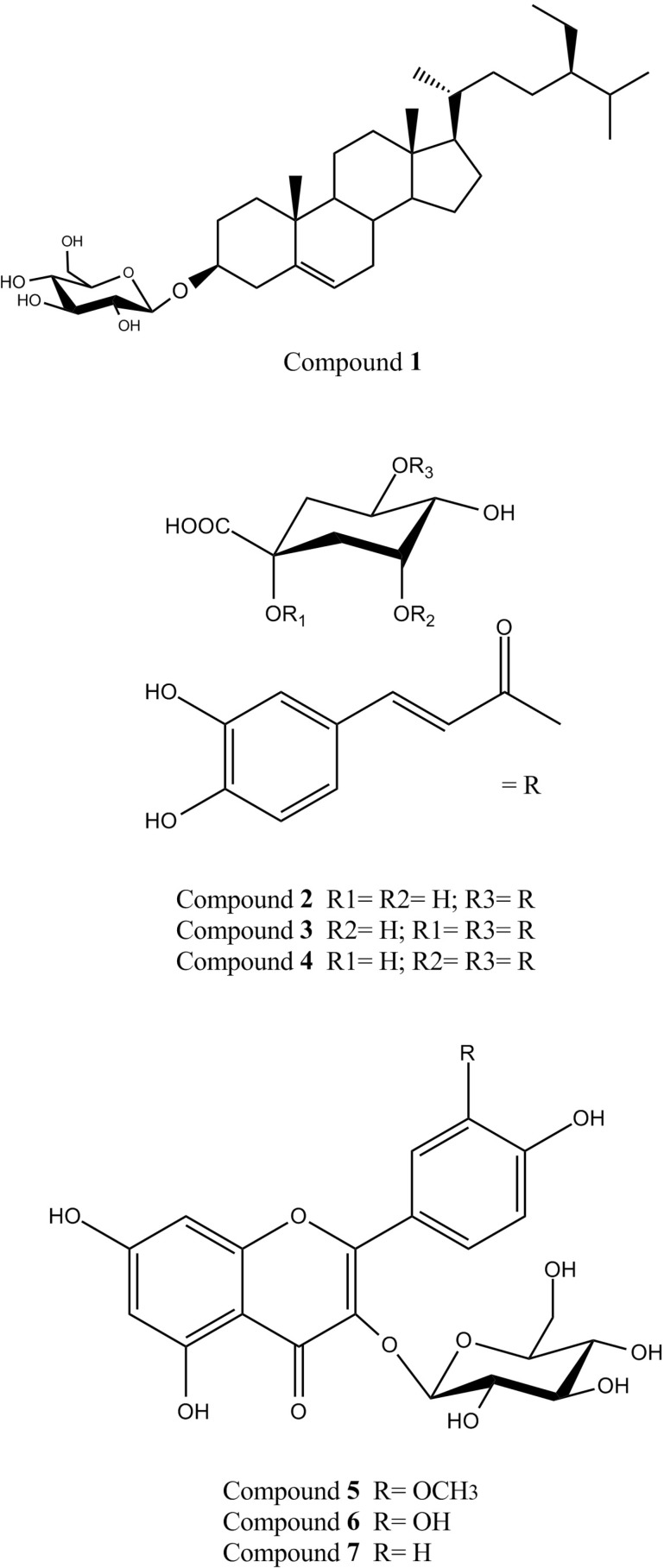
Structures of the isolated compounds from theaerial parts of *D. glabrum.*

Different chromatographic methods on silica gel (normal and reversed-phase) and sephadex LH_20_ columns led to the isolation of 1; β-sitosterol-3-O-β-D-glucopyranoside (daucosterol) (21) and 2; 5-O-caffeoylquinic acid (chlorogenic acid) (22) from the ethyl acetate fraction,together with a mixture of 3; 1,5-di-O-caffeoylquinic acid (cynarin) (23) and4; 3,5-di-O-caffeoylquinic acid (23), 5; isorhamnetin-3-O-β-D-glucopyranoside (24, 26), 6; quercetin-3-O-β-D-glucopyranoside (isoquercetin) (25, 26) and 7; kaempferol-3-O-β-D-glucopyranoside (astragalin) (25, 26) from the methanol fraction of *D. glabrum* aerial parts. The structures of these compounds were established by comparison of their spectroscopic data (NMR and UV) with those published in the literature(Figure 1) (23-28).

A previous study on *D. aucheri* has reported isolation of four exudates methoxylated flavones (salvigenin, nepetin, crisiliol and eupatorin) from its aerial parts (29). There is also a report on isolation of three new sesquiterpene derivatives (kopetdaghins A-C) along with a known prenylated coumrin, daucosterol and stigmasterol-3-O-glucoside from the aerial parts of *D. kopetdaghense *(30). To our knowledge, present study is the first report on the isolation of compounds 2-7 from *Dorema* genus plants. The results of our phytochemical studies also indicated thatthe major phloroglucinol glycosides reported from the roots of *D. hyrcanum* and *D. aitchisonii* (hyrcanoside, pleoside and echisoside), were not present (at least at high levels) in the aerial parts of *D. glabrum *(31, 32).

A review of the literature demonstrated that the isolated compounds from *D.glabrum* aerial parts have been documented for their various biological properties (23, 27, 33-38). Despite of poor antioxidant activity of daucosterol (1), it has been found to possess anti-inflammatory, anti-neoplastic, and immunomodulatory activities (23). Mono- and di-caffeoylquinic acid derivatives have also been considered for their beneficial health effects such as antioxidant, hepatoprotective, hypocholesterolemic, anti-inflammatory and anti-PAF activity (33, 34). It could be therefore assumed that these phenolic compounds (2-4) are involved in previously reported hypocholesterolemic effects of *D. glabrum*aerial parts(6). Finally, antioxidant, anti-diabetic and anti-inflammatory properties of the tree isolated flavonol-3-O-glucosides (5-7) have been shown during previous pharmacological investigations (27, 35-38).

The results of free radical scavenging activities of the crude extract, fractions and isolated compounds in DPPH assay were summarized in Table 2. Methanol fraction exhibited the highest level of activity (IC_50_=53.3±4.7μg mL^-1^) among the tested fractions. Isolated compounds from the methanol fraction (3-7) also showed significant free radical scavenging activity (Table 2). These phenolic compounds (3-7), thus could be considered as responsible for the observed notable antioxidant activity of the methanol fraction. Among the isolated compounds, caffeoylquinic acid derivatives (2-4) were found to possess potent free radical scavenging activity (IC_50_=2.23±7.3 and 2.61±4.8μg mL^-1^) higher than BHT, a synthetic commercial antioxidant(IC_50_= 19.5±0.8 μg mL^-1^). Considering to the recognized important role of antioxidants in disease prevention and health promotion (39), our results introduce *D. glabrum* as a valuable source of natural phenolic antioxidants specially flavonoids and caffeoylquinic acid derivatives.

Occurrences of biologically active compounds in *D. glabrum *aerial parts are indicative of more medicinal potentials of this species and make it an appropriate candidate for further pharmacological and toxicological studies. The results of our study also emphasizes on necessity of conservation of *D. glabrum*, as a valuable genetic resource for bioactive natural products.

**Table 2:** Free radical scavenging activities of the crude extract, fractions and isolated compounds from the aerial parts of *D. glabrum*

**Extract/fractions/compounds**	**IC**_50_ ** value (μg mL** ^-1^ **)** ^a^
Crude extract	68.2 ± 4.1
Petroleum ether fraction	123.1 ± 7.0
Chloroform fraction	94.5 ± 5.2
Ethyl acetate fraction	71.8 ± 2.8
Methanol fraction	48.3 ± 4.7
Daucosterol (**1**)	224.1 ± 8.2
Chlorogenic acid (**2**)	2.23 ± 0.6
Cynarin and 3,5-Di-O-caffeoylquinic acid (**3**,**4**)	2.61 ± 0.4
Isorhamnetin-3-O-β-D-glucopyranoside (**5**)	40.6 ± 3.2
Isoquercetin (**6)**	24.6 ± 3.1
Astragalin (**7**)	43.2 ± 4.2
BHT (Positive control)	19.5 ± 2.8

Note: a Concentration providing 50% inhibition, calculated for each sample from the graph plotting inhibition percentages against concentrations of the sample.
